# Drug-Disease Association Prediction Using Heterogeneous Networks for Computational Drug Repositioning

**DOI:** 10.3390/biom12101497

**Published:** 2022-10-17

**Authors:** Yoonbee Kim, Yi-Sue Jung, Jong-Hoon Park, Seon-Jun Kim, Young-Rae Cho

**Affiliations:** 1 Division of Software, Yonsei University Mirae Campus, Wonju-si 26493, Gangwon-do, Korea; 2 Division of Digital Healthcare, Yonsei University Mirae Campus, Wonju-si 26493, Gangwon-do, Korea

**Keywords:** drug repositioning, drug–disease associations, heterogeneous networks, drug networks, disease networks

## Abstract

Drug repositioning, which involves the identification of new therapeutic indications for approved drugs, considerably reduces the time and cost of developing new drugs. Recent computational drug repositioning methods use heterogeneous networks to identify drug–disease associations. This review reveals existing network-based approaches for predicting drug–disease associations in three major categories: graph mining, matrix factorization or completion, and deep learning. We selected eleven methods from the three categories to compare their predictive performances. The experiment was conducted using two uniform datasets on the drug and disease sides, separately. We constructed heterogeneous networks using drug–drug similarities based on chemical structures and ATC codes, ontology-based disease–disease similarities, and drug–disease associations. An improved evaluation metric was used to reflect data imbalance as positive associations are typically sparse. The prediction results demonstrated that methods in the graph mining and matrix factorization or completion categories performed well in the overall assessment. Furthermore, prediction on the drug side had higher accuracy than on the disease side. Selecting and integrating informative drug features in drug–drug similarity measurement are crucial for improving disease-side prediction.

## 1. Introduction

The development of new drugs is a time-consuming, expensive, and high-risk task. The drug release process requires extensive effort and investment from drug design to preclinical development, clinical trials, and regulatory approval [1]. The average development period for new drugs is 13.5 years, costing more than USD 1.8 billion [2]. Although investments in drug research are steadily increasing, the number of new drugs entering the market is decreasing. Therefore, increasing the success rate of drug research is crucial in saving time and cost. Recently, drug repositioning, which involves the identification of new therapeutic indications for already approved drugs, has gained attention as it considerably reduces the time and cost of discovering new drugs [3]. In addition, existing drugs have already been demonstrated to be safe via clinical trials. Several successful instances of drug repositioning have occurred in the past decades; for example, sildenafil was initially developed to treat coronary artery disease, but was repositioned to treat erectile dysfunction [4]. During the COVID-19 pandemic, the search for effective therapeutic agents was urgent and remdesivir was successfully repositioned to treat COVID-19 [5].

Although a majority of drug repositioning efforts have been determined by clinical observations, computational methods have recently been proposed to predict candidate drugs for repositioning effectively [6,7]. They are also scalable to genome-wide data for drugs, diseases, and genes or proteins from multiple aspects. Most computational methods leverage networks structured by the relationships among biomedical entities and facilitate systematic analysis of the networks [8,9,10]. In this review, we provide a comprehensive overview of the recent network-based approaches for computational drug repositioning.

In the early stage, computational drug repositioning was primarily focused on the discovery of interactions between a drug and its molecular targets. Evidence of drug–target interactions (DTIs) provides significant clues for drug repositioning based on the underlying assumption that multiple drugs interact with multiple targets [11]. Most previous DTI prediction methods explored networks, such as a drug–target bipartite network [12,13], to infer new targets for each drug. Various network-based methods have been proposed for DTI prediction until recently [14,15,16]. However, because those methods have already been surveyed by many previous articles [17,18,19,20,21], we narrowed down the scope in this review to predicting associations between drugs and diseases only.

Recent studies that aimed to identify genes causing a particular disease revealed an increasing trend towards network-based analysis. Networks have become essential tools to prioritize genes for each disease [22]. Numerous network-based methods have been proposed for disease–gene association prediction [23,24]. They assume that genes causing the same disease are located close to each other in a network. However, this review does not include disease–gene association prediction methods. This review aims to provide a summary of the recent network-based approaches of drug–disease association prediction for computational drug repositioning and compare their predictive accuracy equitably using uniform datasets.

Network-based methods for drug–disease association prediction mainly involve heterogeneous networks, constructed using diverse features of drugs and diseases. Some of the methods adopt genetic information, such as protein–protein interactions (PPIs), DTIs, and disease–gene associations. The integrated dataset is expanded to a drug–disease–gene heterogeneous network. A homogeneous network consists of a single type of node and their connections, whereas a heterogeneous network is composed of two or more types of nodes and their connections. For example, a heterogeneous drug–disease network G=(V,E) can be created by connecting between homogeneous drug and disease networks, $\left(V_{drug},E_{drug-drug}\right)$ and $\left(V_{disease},E_{disease-disease}\right)$, represented as

$$V=V_{drug}\cup V_{disease}$$

and

$$E=E_{drug-drug}\cup E_{disease-disease}\cup E_{drug-disease}.$$

Similarly, a heterogeneous drug–disease–gene network $G^{\prime }=\left(V^{\prime },E^{\prime }\right)$ can be constructed by additional links between *G* and a homogeneous gene network $\left(V_{gene},E_{gene-gene}\right)$ as

$$V^{\prime }=V_{drug}\cup V_{disease}\cup V_{gene}$$

and

$$E^{\prime }=E_{drug-drug}\cup E_{disease-disease}\cup E_{gene-gene}\cup E_{drug-disease}\cup E_{drug-gene}\cup E_{disease-gene}.$$

Network-based drug–disease association prediction methods are divided into three major categories: graph mining, matrix factorization or completion, and deep learning. The methods in the first category apply graph mining algorithms, such as random walk, network propagation, and path search, to detect putative associations between unlinked drug–disease pairs in a heterogeneous network. The methods in the second category use matrix factorization or matrix completion to compute predictive scores for drug–disease pairs. Homogeneous and heterogeneous networks are represented in the form of matrices. Matrix factorization is a collaborative filtering technique that converts the entries of sparse matrices, such as drug–disease associations, into predictive scores. The methods in the third category adopt deep learning algorithms, such as autoencoders and graph convolutional networks (GCN), to build a predictive model using network features.

In this article, we survey existing network-based drug repositioning methods in the three categories, and experimented with 11 selected methods to compare their predictive accuracy. Our experiments were conducted using two uniform datasets in two different approaches, respectively: to predict associations on the drug side (i.e., to predict diseases associated with each new drug) and predict associations on the disease side (i.e., predict drugs associated with each new disease). Finally, their performance was analyzed using several evaluation metrics.

## 2. Review of Network-Based Drug-Repositioning Approaches

**Figure 1:**
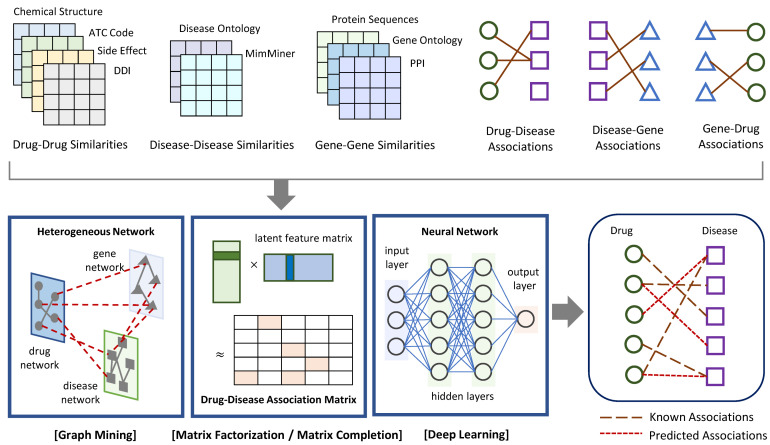
Schematic view of the general process of network-based approaches to predict drug–disease associations. They take a heterogeneous network including drug–drug, disease–disease, and gene–gene similarities as well as known drug–disease, drug–gene, and disease–gene associations as input. They apply graph mining algorithms, or matrix factorization and completion techniques, or deep learning algorithms to return predicted drug–disease associations as output.

In this section, network-based approaches for drug–disease association prediction were introduced from the following three categories: graph mining, matrix factorization or completion, and deep learning. Figure 1 exhibits the general process of the methods in the three categories. They take drug–drug, disease–disease, and gene–gene similarities as well as drug–disease, drug–gene, and disease–gene associations as input, and predict new drug–disease associations as output. Table 1 lists recently proposed network-based methods and the main techniques that support their algorithms. This list also includes data sources to construct the drug network (i.e., drug–drug similarities), disease network (i.e., disease–disease similarities), and gene network (i.e., gene–gene similarities) for each method.

### 2.1. Graph Mining Algorithms

#### 2.1.1. Heterogeneous Network Clustering

As the earliest study of drug-disease association prediction, Wu et al. [25] proposed to apply graph clustering to a heterogeneous network including drugs and diseases. Drugs and diseases were extracted from the KEGG Medicus database [44] and drug–disease associations were scored by the Jaccard coefficient of commonly associated genes. Drug–drug and disease–disease similarities were also measured by the Jaccard coefficients of shared features such as biological processes, pathways, and phenotypes. Finally, they applied a clustering algorithm, ClusterONE [45], to the weighted heterogeneous network for predicting new drug–disease associations.

#### 2.1.2. TL_HGBI

Most drug–disease association prediction methods only use drug and disease data. However, Wang et al. [26] further used genetic relationships because the therapeutic effect on a disease is achieved through a combination involving disease–related molecular targets. The proposed model, triple-layer heterogeneous graph-based inference (TL_HGBI), merges network-based drug repositioning and DTI prediction into a unified framework. Drug, disease, and gene networks are constructed based on drug–drug, disease–disease, and gene–gene similarities, and integrated into a three-layer heterogeneous network by drug–disease associations and DTIs. The information propagation algorithm iteratively updates the weights of drug–disease, drug–gene, and disease–gene pairs on the heterogeneous network to infer new drug–disease associations.

#### 2.1.3. DrugNet

DrugNet [27] executes an information propagation algorithm on drug, disease, and protein similarity networks using ProphNet [46], a general network-based prioritization tool. This method searches for paths from the drug network to the disease network and executes propagation within a network and between networks alternately through such paths. A random walk with restart (RWR) algorithm is adopted for propagation within a network. The two propagation processes are repeated until the walker reaches an adjacent node of the disease network. Finally, the predictive score of each drug–disease pair is computed based on the propagated quantities.

#### 2.1.4. MBiRW

Luo et al. [28] suggested the measurement of drug–drug and disease–disease similarities using the features of drugs and diseases as well as known drug–disease associations. The proposed method, similarity measures and bi-random walk (MBiRW), updates drug and disease networks based on drug–disease associations in two steps. First, the method identifies informative pairs of drugs that are associated with common diseases and adopts a logistic function to shrink the similarities between non-informative pairs to zero and enlarge the similarities between informative pairs. Second, the method creates a drug-sharing network that is weighted by the number of common diseases and applies clustering to the network assuming that two drugs are similar if they share common diseases or if other drugs that share diseases with them exist simultaneously. The drug network is updated by increasing the similarities between drugs belonging to the same cluster. The disease network is updated likewise. Using the resultant networks, this method performs bi-random walks based on the following equations:

(1) $$X_{t}^{l}=\alpha \times R\times X_{t-1}+\left(1-\alpha \right)\times M$$

(2) $$X_{t}^{r}=\alpha \times X_{t-1}\times D+\left(1-\alpha \right)\times M\;$$

where $X_{t}^{l}$ and $X_{t}^{r}$ represent the predicted drug-disease associations walking on the drug and disease networks, respectively, at time *t*. *R* and *D* are the drug and disease networks, respectively, *M* indicates the drug–disease association matrix, and α represents a parameter that regulates the importance between networks. Random walk is performed separately on the drug and disease networks. The final predictive scores are computed using the average of the two probabilities.

#### 2.1.5. TP-NRWRH

Liu et al. [29] proposed a two-pass network-based RWR on the heterogeneous network (TP-NRWRH) which is an advanced version of the NRWRH proposed by Chen et al. [47]. In TP-NRWRH, a random walk is performed in two phases, namely a drug-centric random walk and disease-centric random walk, to detect candidate associations. The two results are combined using the mean value to compute the final predictive score for each association. During the random walk, this method applies the jump probability and restart probability for the walker to move to a different type of node and the start node, respectively.

#### 2.1.6. DR-IBRW

Wang et al. [30] proposed drug repositioning based on individual bi-random walk (DR-IBRW). Unlike MBiRW [28], which uses a fixed walk length, this algorithm uses an individual walk length as all nodes contribute differently to transferring information in the heterogeneous network. DR-IBRW measures drug–drug and disease–disease similarities based on their chemical fingerprints and symptoms, respectively. Similar to MBiRW, this method applies a logistic function and graph clustering algorithm. DR-IBRW also adopts the Gaussian interaction profile (GIP) kernel to extract more similarities that are combined with existing similarities. Moreover, it calculates the modified Jaccard index on the bipartite network to weight drug–disease associations. A bi-random walk with restart is then performed on the heterogeneous network. A random walker takes a drug as the starting node and traverses other disease nodes. Similarly, a random walker takes a disease as the starting node and traverses other drug nodes. This method eventually integrates the association confidence from the perspective of the disease similarity and drug similarity networks.

#### 2.1.7. EMP-SVD

Wu et al. [31] proposed a path-based model on a heterogeneous network, named ensemble meta paths and singular value decomposition (EMP-SVD). Unlike other network-based approaches, this method does not use drug–drug or disease–disease similarities because the similarity scores for the same item may be significantly different according to the features used. This model handles the associations of drug–disease, disease–protein, and drug–protein only. After integrating these associations into a single heterogeneous network, the method searches for five meta-paths as features.

- Drug → (treats) → Disease
- Drug → (binds to) → Protein → (causes) → Disease
- Drug → (binds to) → Protein → (binds to) → Drug → (treats) → Disease
- Drug → (treats) → Disease → (treated by) → Drug → (treats) → Disease
- Drug → (treats) → Disease → (caused by) → Protein → (causes) → Disease

This model constructs a commuting matrix for each meta-path, and obtains latent features using SVD, which are trained by a Random Forest classifier, an ensemble model that avoids overfitting by creating multiple decision trees. Because current association datasets contain positive and unknown data, not negative, this model selects negative samples with a lower chance of associations from unknown pairs. If a drug and a disease are linked to common proteins, this pair is excluded from the negative data.

#### 2.1.8. BGMSDDA

Xie et al. [32] proposed bipartite graph diffusion with multiple similarity integration for drug–disease association prediction (BGMSDDA). This method reconstructs a drug–disease association matrix using the weighted k-nearest known neighbors algorithm. Similar to DR-IBRW [30], this method measures similarities by combining the GIP kernel with those processed by the linear neighborhood similarity algorithm. Thereafter, bipartite graph diffusion is performed. Each node is assigned a weight according to the degree of drugs and pre-processed matrices, and the initial resource spreads to its associated disease nodes through the degree of each drug. Similarly, the initial resource spreads to associated drug nodes through the degree of each disease. Finally, the associations are predicted based on the resultant scores from graph diffusion.

### 2.2. Matrix Factorization or Matrix Completion

#### 2.2.1. DRRS

Luo et al. [33] proposed a drug repositioning recommender system (DRRS) that completes the matrix representing the drug–disease heterogeneous network through rank minimization as follows:

(3) $$\min rank\left(X\right)\;\;\;s.t.\;P_{\Omega }\left(X\right)=P_{\Omega }\left(M\right)$$

where *M* and *X* denote the original and predicted matrices, respectively, and $P_{\Omega }\left(M\right)$ is the set of indices for all known associations in *M*. This method used a single matrix as a heterogeneous network by combining drug–disease associations, drug-drug similarities, and disease–disease similarities. As minimizing the rank of the matrix, which is known as an NP-hard problem, is difficult to solve, the problem was changed to minimize the sum of its singular values, which is also known as its nuclear norm. To determine the optimal matrix rank *r*, this algorithm runs on a random sample, 10% of drug–disease associations, and observes the area under the ROC curve (AUC) with increasing *r*. Finally, this algorithm runs on the entire dataset with selected values of *r* to predict drug-disease associations.

#### 2.2.2. OMC

Exploring a large heterogeneous network for drug–disease association prediction is computationally complex. Yang et al. [34] proposed a matrix completion method, named overlap matrix completion (OMC), to handle two networks for drugs and diseases separately, instead of building a single heterogeneous network. OMC is divided into OMC2 and OMC3 according to the number of knowledge types used.

In OMC2, the drug network consists of drug–drug similarities and drug–disease associations. As a pre-processing step, KNN is performed on the association matrix to reduce sparsity. The updated association matrix is combined with the drug–drug similarity matrix to obtain a block adjacency matrix. The same process is applied to the disease network. The BNNR algorithm [48] is then applied to the drug- and disease-side block adjacency matrices, separately. BNNR performs matrix completion through nuclear norm minimization.

(4) $$\min_{X}{\left||X|\right|}_{*}+\frac{\alpha }{2}\left|\right|P_{\Omega }\left(X\right)-P_{\Omega }\left(M\right){\left|\right|}_{F}^{2}$$

where *M* is the heterogeneous network as input, *X* is the predicted network, and ${\left||X|\right|}_{*}$ is the nuclear norm of *X*. As shown in the equation, this model minimizes the nuclear norm for drug–disease association prediction. The regularization term guarantees predictive scores in the range of 0 to 1. Drug-disease associations are eventually predicted based on the mean of the two results.

OMC3 is an extension of OMC2, that uses additional information regarding drug–protein and disease–protein associations. In OMC3, the drug-side block adjacency matrix is constructed using not only drug–disease associations, but also drug-protein associations. The disease-side block adjacency matrix is constructed likewise.

#### 2.2.3. DRIMC

Zhang et al. [35] proposed drug repositioning by Bayesian inductive matrix completion (DRIMC), which utilizes the features of drugs and diseases as side information for matrix factorization. To construct a drug similarity network, this method integrates chemical structure, target domain, and target annotation similarities and GIP kernel. To construct a disease similarity, this method integrates the disease semantic similarity, and GIP kernel. For the integration of similarities, the method adopts the similarity network fusion (SNF) technique [49]. The drug and disease feature vectors are filled with the similarity values of their k-nearest neighbors on their indices. Logistic matrix factorization is performed to update the latent factor matrices of drugs and diseases. The two latent factor matrices are finally multiplied to generate predictive scores for candidate drug–disease associations.

#### 2.2.4. MSBMF

Yang et al. [36] proposed multi-similarities bi-linear matrix factorization (MSBMF) to predict new indications by incorporating multiple similarity matrices. This method applies drug–drug and disease–disease similarities to feature matrices instead of the latent feature vectors of drugs and diseases on matrix factorization as follows:

(5) $$\min_{U,V}\frac{1}{2}\left|\right|P_{\Omega }\left(UV^{T}-M\right){\left|\right|}_{F}^{2}+\frac{\alpha_{1}}{2}{\left(||U|\right|}_{F}^{2}+{\left||V|\right|}_{F}^{2})+\frac{\alpha_{2}}{2}(||D-UU^{T}{\left|\right|}_{F}^{2}+||R-VV^{T}{\left|\right|}_{F}^{2})$$

where *U* and *V* are the feature matrices and *D* and *R* are the similarity matrices for diseases and drugs, respectively. *M* represents the drug–disease association matrix. In this method, multiple similarities can be used in a concatenated form. For example, a disease similarity matrix *D* is substituted by $D_{m}$ as the phenotypic similarity $D_{ph}$ concatenated with ontological similarity $D_{do}$, which can be described by $D_{m}=\left[D_{ph},D_{do}\right]$. This method optimizes the feature matrix of drugs and diseases, matrices with latent features representing the disease and drug similarity, and an auxiliary matrix to predict drug–disease associations. The alternating direction method of multipliers (ADMM) framework [50], which optimizes each variable while fixing the other variables, is applied to this method.

#### 2.2.5. NTD-DR

Jamali et al. [37] proposed non-negative tensor decomposition for drug repositioning (NTD-DR), which uses pairwise associations of drug–disease, drug–target, and target–disease to construct a three-dimensional association tensor and decompose it into three-factor matrices. The objective function of tensor decomposition is as follows:

(6) $$\min_{A,B,C}L\left(A,B,C\right)=\min_{A,B,C}\left(L_{T}\left(A,B,C\right)+L_{S}\left(A,B,C\right)+L_{A}\left(A,B,C\right)\right)\;$$

where *A*, *B*, and *C* are non-negative factor matrices; L is the least square criterion; $L_{T}$ is the function for third-order tensor decomposition; $L_{S}$ is the function for similarities of drug–drug, target–target, and disease–disease; and $L_{A}$ is the function for drug–target, drug–disease, and target–disease pairwise associations. When the factor matrices *A*, *B*, and *C* converge, the tensor is reconstructed by integrating the drug, target, and disease similarity information. Accordingly, this method predicts drug–target–disease triplet associations and their pairwise associations.

### 2.3. Deep Learning

#### 2.3.1. deepDR

Zeng et al. [38] developed a network-based deep-learning algorithm, deepDR, for computational drug repositioning. This method captures the complex and nonlinear structures of multiple similarity networks. Briefly, the RWR algorithm is applied to each drug-related similarity network to convert the topology structure into a probabilistic matrix, which is transformed into a positive point-wise mutual information (PPMI) matrix [51]. Multiple PPMI networks are provided as the input of a multi-modal deep autoencoder (MDA) to fuse into common features of multiple networks. These low-dimensional drug features are extracted from the middle layer of MDA. In deepDR, these common features are used to solve the sparsity problem in the association matrix. Both the common features and drug–disease associations are encoded and decoded into the inference network and generation network of the collective variable autoencoder (cVAE) [52] to infer new indications.

#### 2.3.2. ANMF

Yang et al. [39] proposed additional neural matrix factorization (ANMF), an approach combining the techniques of matrix factorization and autoencoders. Inspired by the additional denoising autoencoder model, the hidden features of drugs and diseases are extracted using the following procedure. First, the method adds Gaussian noise to the similarity and association matrices and denoises the noisy similarity and association matrices using an autoencoder. Thereafter, the hidden layer of the autoencoder is used as the hidden features. Finally, the hidden features of drugs and diseases are multiplied and the predicted values are generated as follows:

(7) $${\hat{r}}_{i,j}=F_{out}\left(h^{T}\left(U_{i}⊙V_{j}\right)\right)$$

where $U_{i}$ and $V_{j}$ represent the hidden features of drug *i* and disease *j*, respectively, extracted by this model, ⊙ is the element-wise product, *h* represents the weight parameter, $F_{out}$ represents an arbitrary activation function, and ${\hat{r}}_{i,j}$ denotes the predicted values. During back-propagation, the mean square error is employed as the loss function of the autoencoder and binary-entropy is employed as the loss function of prediction. The loss functions are then combined and multiplied by the hyperparameters.

#### 2.3.3. NEDD

Zhou et al. [40] proposed a neural network-based method for learning network representation vectors (NEDD). This method adopts a network embedding technique, called HIN2vec [53], to create vectors. HIN2vec is similar to node2vec [54] but considers the meta-paths representing the sequences of node types and/or edge types. Because the length of the meta-paths is greater than or equal to 1, this method can extract more information regarding network features. HIN2vec learns whether two nodes are linked, as follows:

(8) $$P\left(r|x,y\right)=sigmoid\left(\sum W_{X}^{\prime }\vec{x}⊙W_{Y}^{\prime }\vec{y}⊙f_{01}\left(W_{R}^{\prime }\vec{r}\right)\right)\;$$

where *x* and *y* are nodes, *r* is the path between *x* and *y*; $\vec{x}$, $\vec{y}$, and $\vec{r}$ denote one-hot vectors for *x*, *y*, and *r*; $f_{01}$ represents a regularization function; ${W\prime }_{X}\vec{x}$, ${W\prime }_{Y}\vec{y}$, and $f_{01}\left({W\prime }_{R}\vec{r}\right)$ are the latent vectors for *x*, *y*, and *r*; and ⊙ is the element-wise product. Finally, NEDD conducts a Random Forest classifier to predict drug–disease associations.

#### 2.3.4. SNF-NN

Jarada et al. [41] proposed a model applying the SNF and neural networks (SNF-NN), which integrates similarities by SNF and applies neural networks to drug–disease association prediction. First, this method quantifies the similarity of each drug pair or disease pair using shared characteristics and the GIP kernel. Thereafter, the similarities are integrated by SNF like DRIMC. Finally, the method constructs a neural network that takes the integrated similarities as input and outputs the predicted associations. The neural network is structured by a fully connected feed-forward multi-layer perceptron network containing an input layer, one or more hidden layers, and an output layer. The hyperparameters of the neural network model are tuned by nested cross-validation. This model is initialized by the He initialization algorithm and trained by the Adam optimization algorithm.

#### 2.3.5. SAEROF

Jiang et al. [42] proposed a model that adopts a sparse autoencoder (SAE) to extract valid features from sparse data and applies the Rotation Forest to drug–disease association prediction (SAEROF). First, this model computes the GIP kernel for drug–drug and disease–disease similarities if the GIP kernel produces non-zero values. If the association between a disease and a drug is unknown, the GIP kernel produces a zero. In this case, the structural similarity of drugs or the semantic similarity of diseases is computed. Thereafter, SAE is applied to extract the features. This technique introduces a penalty for learning the sparse functions. Finally, the Rotation Forest classifier [55], an ensemble model that trains the rotating feature-sets extracted by Principal Component Analysis, is applied to learn features and predict associations.

#### 2.3.6. LAGCN

Yu et al. [43] proposed a layer attention graph convolutional network (LAGCN), which adopts GCN layers for drug–disease association prediction. The input adjacency matrix *G* is set as

(9) $$G=\left[\begin{array}{cc} \mu ∼R & M\\ M^{T} & \mu ∼D \end{array}\right]$$

where μ is a penalty factor to control the similarity contribution in the GCN, *R* denotes the normalized drug similarity matrix, *D* denotes the normalized disease similarity matrix, and *M* is the drug–disease association matrix. The GCN iteratively updates embeddings through the layers. Owing to the inconsistency with the contributions of embeddings at different layers, this model applies an attention mechanism that combines the embeddings to obtain final embeddings of drugs and diseases. Decoder $A^{\prime }$ eventually reconstructs the drug-disease association matrix as

(10) $$A^{\prime }=sigmoid\left(H_{R}W^{\prime }H_{D}^{T}\right)$$

where $H_{R}$ and $H_{D}$ are the final embeddings of drugs and diseases, respectively, and $W^{\prime }$ is a trainable matrix. Because the number of known associations is markedly smaller than the number of unknown data, this model also adopts weighted cross-entropy as a loss function to reduce the impact of data imbalance.

## 3. Experiments

For performance evaluation and comparison, 11 drug-disease association prediction methods were selected across three categories: MBiRW, TP-NRWRH, DR-IBRW, and BGMSDDA as graph-mining algorithms; DRRS, OMC, DRIMC, and MSBMF as matrix factorization or completion; and deepDR, ANMF and LAGCN as deep learning algorithms. We attempted to choose evenly from underlying methods to state-of-the-art methods for each category. The selected methods were executed using the uniform datasets under the same experimental conditions for an equitable comparison of their prediction results. In this section, we introduce the datasets used in our experiments and discuss the experimental settings.

### 3.1. Experimental Data

To construct a drug–gene–disease heterogeneous network, the following steps were employed. First, drug–drug, disease–disease, and gene–gene similarities were computed. For our experiment, we used two different drug–drug similarity measures; one was based on their chemical structures (named network-CS) and the other was based on the ATC codes (named network-ATC). Disease–disease and gene–gene similarities were measured by a semantic similarity metric using Human Phenotype Ontology (HPO) [56] and Gene Ontology (GO) [57] annotation datasets, respectively. Next, a tripartite graph was constructed using drug–disease, drug–gene and disease–gene associations. Table 2 shows the numbers of drugs, diseases, genes, and edges (i.e., associations) between them in the tripartite graph that was used in our experiment. Finally, the tripartite graph was merged with the drug–drug, disease–disease, and gene–gene similarities to create a single weighted heterogeneous network. The details of the datasets are discussed in the following subsections.

#### 3.1.1. Drug Network

The drug dataset was extracted from DrugBank5.0 [58]. DrugBank is a comprehensive online database including not only FDA approved drugs but also experimental drugs undergoing approval procedures. We measured similarities for both approved drugs and experimental drugs because drug repositioning involves not only finding new applications of the drugs being used, but also finding new medicinal effects of failed drugs due to lack of clinical efficacy. This database contains diverse features of drugs, such as their chemical structures and pharmaceutical information, and drug targets, such as their sequences, structures, and pathways. In our experiment, drug–drug similarities were measured using two different features: chemical structures and ATC codes.

First, similarities between drug structures were measured using simplified molecular-input line-entry specification (SMILES) [59]. SMILES is a line notation system that describes the structures of chemical compounds using short ASCII strings. For example, the pyruvic acid of DrugBank ID DB00119 is expressed as CC(=O)C(O)=O in the SMILES format. The network comprised 11,219 drugs that had SMILES structure information, among those from DrugBank. We used CDK [60] to obtain fingerprints of the drugs in the SMILES format, and finally computed Tanimoto similarity scores for all drug pairs.

Next, similarities between drugs were measured based on their ATC codes from DrugBank. The Anatomic Therapeutic Chemical (ATC) classification system [61] is a system to classify drugs in a hierarchy of five levels: the anatomical and pharmacological group at the 1st level, the main therapeutic group at the 2nd level, the therapeutic and pharmaceutical subgroup at the 3rd level, the chemical, therapeutic, and pharmacological group at the 4th level, and the chemical substances at the 5th level. Assuming that two drugs have similar features if they are classified with the same code at each level, the similarity is quantified as follows:

(11) $$sim_{atc}\left(d_{i},d_{j}\right)=\frac{S\left(ATC_{d_{i}}\right)\cap S\left(ATC_{d_{j}}\right)}{S\left(ATC_{d_{i}}\right)\cup S\left(ATC_{d_{j}}\right)}$$

where $ATC_{d_{i}}$ indicates the ATC code of drug $d_{i}$ and $S({ATC}_{d_{i}})$ represents a set of codes from all levels of $ATC_{d_{i}}$. It is noted that a drug may have multiple ATC codes; for example, the alteplase of DrugBank ID DB00009 has two ATC codes, B01AD02 and S01XA13. Thus, the similarity between drugs $d_{i}$ and $d_{j}$ was computed as the average similarity of all ATC code pairs from $d_{i}$ and $d_{j}$.

#### 3.1.2. Disease Network

Disease–disease similarities were measured by a semantic similarity metric using annotation data from HPO [56], a comprehensive phenotype ontology. HPO consists of terms in the context of phenotypic abnormalities. It is structured by linking term pairs in a parent-child relationship with a directed edge and is represented as a directed acyclic graph. The HPO includes extensive clinical annotations of human diseases that are provided by OMIM [62], OrphaNet [63], and DECIPHER [64] databases. In our experiments, diseases were extracted from the HPO annotations provided by OMIM. This dataset contained 6465 diseases after those with the evidence code IEA were excluded. To calculate similarities between diseases, a semantic similarity metric integrating annotation- and term-based algorithms was applied, as proposed in [65]. The semantic similarity between two HPO terms is calculated as follows:

(12) $$sim_{T}\left(C_{1},C_{2}\right)=\frac{\sum_{C_{i}\in A_{t}\left(C_{1}\right)\cap A_{t}\left(C_{2}\right)}\log P\left(C_{i}\right)}{\sum_{C_{j}\in A_{t}\left(C_{1}\right)\cup A_{t}\left(C_{2}\right)}\log P\left(C_{j}\right)}$$

where $C_{1}$ and $C_{2}$ are HPO terms, $A_{t}\left(C\right)$ denotes the set of ancestor terms of *C* in HPO, $P(C_{i})$ indicates the ratio of the number of annotations to term $C_{i}$ over the number of annotations to all terms in the ontology, and $-\log P(C_{i})$ refers to the information content of $C_{i}$. Accordingly, the semantic similarities of the term pairs annotating two diseases $d_{1}$ and $d_{2}$ are aggregated to the similarity between $d_{1}$ and $d_{2}$ by best-match averaging, which returns the average of the best matching semantic similarity scores for each term as follows:

(13) $$sim\left(d_{1},d_{2}\right)=\frac{\sum_{C_{i}\in T\left(d_{1}\right)}\max_{C_{j}\in T\left(d_{2}\right)}sim_{T}\left(C_{i},C_{j}\right)+\sum_{C_{j}\in T\left(d_{2}\right)}\max_{C_{i}\in T\left(d_{1}\right)}sim_{T}\left(C_{i},C_{j}\right)}{|T\left(d_{1}\right)|+|T\left(d_{2}\right)|}$$

where T(d) is the set of terms to which the disease *d* is annotated. Similarities for all disease pairs were computed by Equation (13).

#### 3.1.3. Gene Network

Protein-protein interactions (PPIs) from BioGrid [66] were employed for gene-gene similarities in our experiment. After removing duplicate interactions and self-loops in the PPI data set, we measured similarities for all interacting protein pairs using annotation data from GO [57] which is the most widely referenced ontology database for functional genomic studies. This database consists of terms as the unified representation of the features of genes and gene products spanning three sub-ontologies: biological processes, molecular functions, and cellular components. Similar to HPO, GO is structured by parent-child relationships for linked term pairs and provides comprehensive annotations of genes and gene products to the terms. To measure the similarity of each PPI, we applied Equations (12) and (13) to the terms in the sub-ontologies of biological processes and molecular functions. We removed annotations with the evidence code IEA to ensure quality of the resultant similarity scores. Finally, a gene network weighted by semantic similarity was obtained.

#### 3.1.4. Associations

The most widely used drug–disease association datasets in previous drug repositioning studies include Fdataset as a gold standard from [67], and Cdataset [28], which is an expanded version of Fdataset by adding clinically validated drug–disease associations from [27]. In our experiment, drug–disease associations in Cdataset were used to create heterogeneous networks. As depicted in Table 2, network-CS, which was formed by drug–drug similarities based on chemical structures, contained 1728 drug–disease associations between 615 drugs and 285 diseases, whereas network-ATC based on ATC codes contained 1681 drug–disease associations between 593 drugs and 282 diseases. Since network-CS and network-ATC included different numbers of distinct drugs, the number of drug-disease associations in the two networks also changed.

Drug–gene associations were extracted from the Comparative Toxicogenomics Database (CTD) [68] using CAS numbers and gene symbols. As a result, network-CS contained 170,652 drug–gene associations between 450 drugs and 14,430 genes whereas network-ATC contained 167,535 drug–gene associations between 437 drugs and 14,430 genes.

Finally, disease–gene associations were extracted from OMIM, which provides information regarding the relationship between phenotypes and genotypes. Excluding the associations with diseases and genes that do not exist in the disease and gene networks, a total of 390 disease–gene associations between 180 diseases and 323 genes were obtained.

### 3.2. Experimental Settings

Drug–disease association prediction results on the drug and disease sides were separately evaluated. Association prediction on the drug side indicated the prediction of additional diseases to be treated by each drug, whereas association prediction on the disease side indicated the prediction of drugs with the potential to treat each disease. We attempted 10-fold cross-validation for each side. For a balanced assessment, we evenly divided the folds according to the number of drugs, diseases, and their associations.

Prediction accuracy was compared using the most widely employed evaluation metrics: the AUC and the area under the precision-recall curve (AUPR). The AUC is generally considered the most effective metric for quantifying predictive power. ROC curves were generated by tracing the true positive rate (TPR), which indicates the ratio of correct predictions to real positive cases, and the false positive rate (FPR), which indicates the ratio of incorrect predictions to real negative cases. The AUPR, as a significant measure for classification accuracy, represents the area under the precision-recall curve, which demonstrates the precision changes as recall increases. Recall is the same as TPR, whereas precision is the ratio of correct predictions to positively predicted samples.

(14) $$\mathrm{Precision}=\frac{\mathrm{TP}}{\mathrm{TP}\;+\;\mathrm{FP}}$$

where TP and FP indicate the numbers of true positives and false positives, respectively. However, because drug–disease associations are very sparse, this special condition generally yields a markedly greater FP than TP, resulting in extremely low precision. Therefore, we upgraded precision to resolve the data imbalance issue. The new metric of precision uses TPR and FPR instead of TP and FP as follows:

(15) $$\mathrm{Precision}*=\frac{\mathrm{TPR}}{\mathrm{TPR}\;+\;\mathrm{FPR}}$$

where TPR = TP/(TP+FN) and FPR = FP/(FP+TN). Because TPR and FPR have the same range, the prediction results are assessed properly regardless of data imbalances using this metric. Thus, we employed the modified AUPR, named AUPR*, using this new metric of precision for the comparison of prediction accuracy.

## 4. Results

The drug–disease association prediction performance of the 11 selected methods was assessed using two heterogeneous networks: network-CS and network-ATC. Predictions were tested on the drug and disease sides separately, as previously described. In this section, we compared the predictive accuracy of the 11 selected methods using AUC, AUPR, and AUPR*, which is a modified version of AUPR.

### 4.1. Accuracy Comparison with Network-CS

First, we compared the predictive accuracy when network-CS was used. As discussed in the previous section, 10-fold cross-validation was applied to obtain an entire list of predicted associations. Based on the predicted associations in descending order of their predictive scores, ROC and AUPR curves were plotted by alternating the threshold from the highest predictive score to the lowest. AUC, AUPR, and AUPR* were calculated until all associations were predicted to be positive, i.e., the threshold was the lowest.

Table 3 presents the AUC, AUPR, and AUPR* results for each method. Overall, the methods in the graph mining and matrix factorization or completion categories achieved higher accuracy than those in deep learning. Among the graph mining algorithms, TP-NRWRH performed best on the disease side (i.e., prediction of drug-disease associations for new diseases), and MBiRW and DR-IBRW performed well relatively. Among the matrix factorization or completion methods, OMC and MSBMF performed best, particularly on the drug side (i.e., prediction of drug-disease associations for new drugs). When predictions on the drug and disease sides were compared, most methods had slightly higher AUC and AUPR* on the drug side than the disease side, except MBiRW and TP-NRWRH. This indicates that predicting drug–disease associations for each new drug is more accurate than that for each new disease.

**Figure 2:**
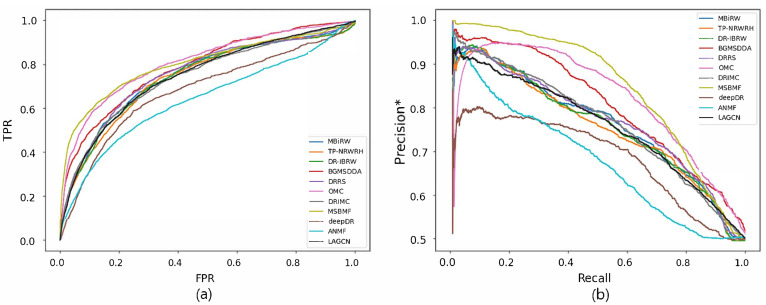
ROC curve (**a**) and precision*-recall curve (**b**) of drug-disease association prediction results for new drugs with network-CS.

**Figure 3:**
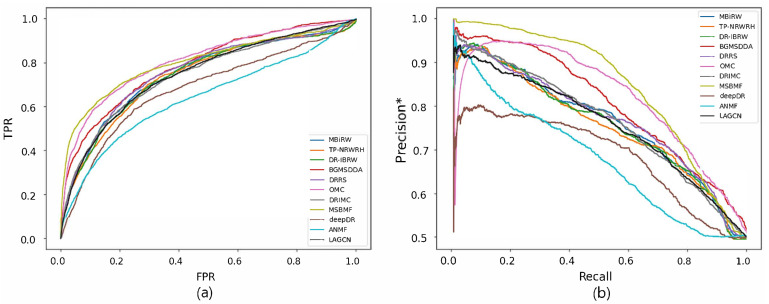
ROC curve (**a**) and precision*-recall curve (**b**) of drug-disease association prediction results for new diseases with network-CS.

Figure 2 and Figure 3 show the ROC and precision*-recall curves for predicting associations on the drug and disease sides, respectively. As MSBMF and OMC showed the best predictive performance on the drug side in Table 3, their TPR increased rapidly (Figure 2a) and precision* decreased slowly as recall increased (Figure 2b). On the other hand, TP-NRWRH and OMC, which had the best performance on the disease side as mentioned in Table 3, showed a rapid increase in TPR (Figure 3a).

### 4.2. Accuracy Comparison with Network-ATC

Next, we compared predictive accuracy when network-ATC was used. AUC, AUPR, and AUPR* results for each method are listed in Table 4. Similar to the results with network-CS in Table 3, the methods in the graph mining and matrix factorization or completion categories achieved higher accuracy than those in deep learning. Among the graph mining algorithms, MBiRW outperformed on the drug side. Among the matrix factorization or completion methods, OMC outperformed on the disease side, whereas DRRS performed well on the drug side. In addition, similar to the results with network-CS, most methods displayed higher AUC and AUPR* on the drug side than the disease side. Finally, when the results in Table 3 and Table 4 were compared, prediction using network-ATC achieved higher accuracy than using network-CS across all selected methods. This indicates that ATC codes might be a better feature than chemical structures to measure drug–drug similarities for drug repositioning.

**Figure 4:**
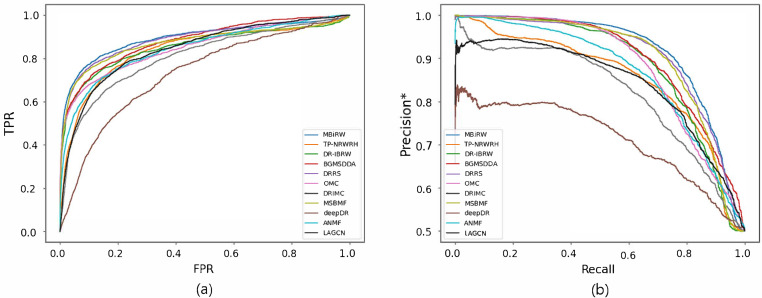
ROC curve (**a**) and precision*-recall curve (**b**) of drug-disease association prediction results for new drugs with network-ATC.

**Figure 5:**
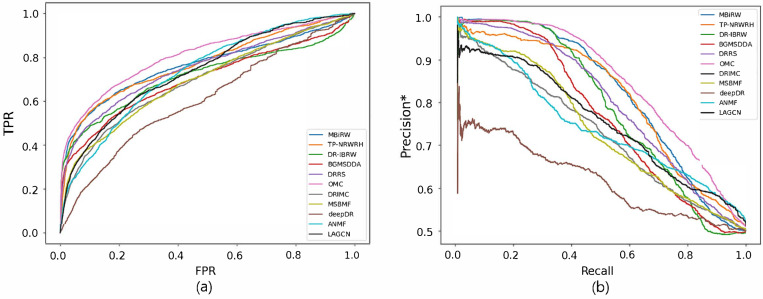
ROC curve (**a**) and precision*-recall curve (**b**) of drug-disease association prediction results for new diseases with network-ATC.

Figure 4 and Figure 5 reveal the ROC and precision*-recall curves for predicting associations on the drug and disease sides, respectively. MBiRW, which had the best predictive performance on the disease side as mentioned in Table 4, showed slightly rapider increase of TPR (Figure 4a) and slightly slower decrease of precision* (Figure 4b) although all the other methods, except deepDR, exhibited relatively good performance in both curves. As OMC showed the best performance on the disease side in Table 4, its TPR increased rapidly (Figure 5a) and precision* decreased slowly as recall increased (Figure 5b).

### 4.3. Robustness Comparison

Drug repositioning algorithms should perform well on incomplete data or noisy data. We assessed the robustness of the selected algorithms by predictive accuracy changes when a certain number of edges were removed from or added to the drug-disease association dataset. For this evaluation, we randomly selected 90% of drug–disease associations in the gold standard Fdataset [67] for training, and predicted the other associations. Next, we added 10% and 20% of random drug–disease pairs to the training dataset. We also removed 10% and 20% of random drug–disease associations from the training dataset.

Predictive accuracy changes by removing and adding drug–disease associations are compared in Table 5. This table shows AUC results on drug–disease association prediction using the gold standard dataset, and the increasing or decreasing rates of the AUC by 10% and 20% of edge removal and addition, respectively. As a result, OMC achieved excellent performance showing the highest accuracy and were robust with incomplete and noisy data. Its predictive accuracy was even increased by removing a certain amount of the drug–disease associations. Overall, the methods in the matrix factorization or completion category were more robust than those in the other groups.

### 4.4. Efficiency Comparison

Because known drug–disease association datasets are constantly updated, drug repositioning experiments may be repeated for every disease newly developed. We evaluated efficiency of the selected algorithms using a genome-scale large heterogeneous network including 3245 drugs from DrugBank, 6322 diseases from HPO annotations, and 98,745 drug-disease associations from CTD. The algorithms were implemented under the specifications of Core i9, DDR4 128GB, and RTX 2080. The elapsed time was measured from reading the input data to storing the prediction results into an output file.

**Figure 6:**
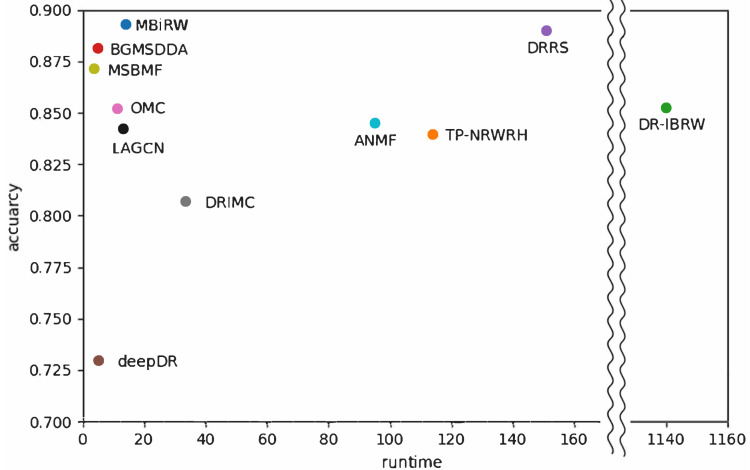
Comparison of efficiency versus accuracy of the selected methods for drug-disease association prediction. Runtime was measured when the selected algorithms were implemented on a genome-scale heterogeneous network.

Figure 6 shows a comparison of runtime on the genome-scale dataset versus predictive accuracy with network-ATC on the drug side shown in Table 4. DR-IBRW in the graph mining category required the longest time to predict associations for all diseases. TP-NRWRH in graph mining, DRRS in matrix completion, and ANMF in deep learning also demanded a long time for prediction, more than 90 h. As a result, DR-IBRW remarkably loses computational efficiency in prediction with a large dataset or a dense network. The time complexity of DR-IBRW is known as $O(n^{4})$ in the worst case.

## 5. Discussion and Conclusions

Drug repositioning, which involves the identification of new indications for existing drugs, delivers substantial benefits to drug development as it is safe and reduces time and cost. In particular, computational drug repositioning methods using networks are promising in that they can efficiently generate candidates. Various computational drug repositioning approaches have recently been introduced. Among network-based methods in the three categories of graph mining algorithms, matrix factorization or completion, and deep learning algorithms, 11 were selected for an unbiased comparison of their predictive accuracy.

In our experiments, drug–drug similarities were measured based on chemical structures and ATC codes, and disease–disease similarities were computed by a semantic similarity metric in an ontology. Moreover, clinically validated drug–disease associations as an extended gold standard dataset, drug–gene associations from CTD, and disease–gene associations from OMIM were integrated with the similarity networks to construct two types of heterogeneous networks. The selected methods predicted drug–disease associations for each drug and each disease, separately. ROC and precision*-recall curves were used to assess the prediction performance of each method. The precision*-recall curve is a modified version of the precision-recall curve, which reflects data imbalance as positive drug-disease associations are sparse in the entire heterogeneous network.

Our experimental results demonstrated that the methods in the categories of graph mining and matrix factorization or completion performed better than those in deep learning. Furthermore, matrix factorization or completion methods, such as OMC, are robust on incompleteness or noise of data. Among the methods based on matrix factorization or completion, OMC exhibited the best predictive performance on both drug and disease sides, and DRRS and MSBMF performed well on the drug side. Among the graph mining algorithms, MBiRW displayed competitive performance on the drug side, whereas TP-NRWRH showed relatively good performance on the disease side. MBiRW and TP-NRWRH have a common process of upgrading the drug and disease similarity networks using known drug–disease associations. Accordingly, prior information involving associations can improve drug–drug and disease–disease similarity scores and result in higher predictive accuracy. Furthermore, OMC, MSBMF, and MBiRW were implemented with very short runtimes. These methods are thus highly pertinent to genome-scale prediction of drug–disease associations.

Next, the selected methods had higher predictive accuracy on the drug side than the disease side. This implies that identifying additional diseases to be treated by a new drug could be more accurate than discovering drugs with the potential to treat a new disease. The higher accuracy on the drug side might be caused because disease–disease similarities are more precise than drug–drug similarities for drug repositioning. We used a comprehensive ontology database HPO and an advanced semantic similarity metric, which has been well-studied with GO, to measure disease–disease similarities. On the other hand, drug–drug similarities were measured by chemical structures (network-CS) and ATC codes (network-ATC) in our experiment. The selected methods had higher predictive accuracy on network-ATC than network-CS. This indicates that ATC codes could be a better feature than chemical structures for drug–drug similarity measurement. However, additional and integrated features are demanded to improve disease-side prediction.

Finally, deep learning algorithms showed lower predictive accuracy than methods from the other groups in the overall assessment. However, when we tested drug–disease association prediction using the genome-scale heterogeneous network which was applied to our efficiency evaluation, accuracy of ANMF and LAGCN increased considerably although this experiment was not relevant to drug repositioning because the dataset included all possible drug–disease associations from CTD. As a result, deep learning algorithms revealed substantially different predictive accuracy depending on the dataset used. Seven hyperparameters were employed for LAGCN as default, and its prediction results changed by a large margin as the hyperparameters changed. Adjusting and optimizing hyperparameters according to the input data would represent key issues in deep learning methods for maintaining consistent accuracy. In diverse domains of bioinformatics, deep learning approaches have already been validated as the most powerful and efficient way to deal with massive amounts of data [69,70].

## Figures and Tables

**Table 1:** List of network-based methods for predicting drug–disease associations in three categories. This list also shows the main algorithms and the features (or tools) to construct drug–drug, disease–disease, and gene–gene similarity networks for each method.

Category	Method	Algorithms	Features (or Tools) Used for Network Construction		
			Drug Network	Disease Network	Gene Network
Graph Mining	Wu et al. [25]	graph clustering	biological processes, pathways, phenotypes	biological processes, pathways, phenotypes	-
	TL_HGBI [26]	propagation	chemical structures, DTIs	MimMiner	protein sequences
	DrugNet [27]	propagation	ATC codes	semantic sim.(DO)	PPIs
	MBiRW [28]	bi-random walk	chemical structures, drug-disease assoc.	MimMiner, drug-disease assoc.	-
	TP-NRWRH [29]	random walk	chemical structures, drug-disease assoc.	MimMiner, drug-disease assoc.	-
	DR-IBRW [30]	bi-random walk	chemical structures, drug-disease assoc.	symptoms, drug-disease assoc.	-
	EMP-SVD [31]	meta-path search	chemical structures	MimMiner	protein sequences
	BGMSDDA [32]	graph diffusion	chemical structures	MimMiner	-
Matrix Factorization / Matrix Completion	DRRS [33]	nuclear norm minimization	chemical structures	MimMiner	-
	OMC [34]	nuclear norm minimization	chemical structures, DTIs	MimMiner, disease-gene assoc.	-
	DRIMC [35]	logistic matrix factorization	chemical structures, target domain, target annotation	MimMiner	-
	MSBMF [36]	bilinear matrix factorization	chemical structures, ATC codes, side effects, DDIs, target profiles	MimMiner, semantic sim.(DO)	-
	NTD-DR [37]	tensor decomposition	chemical structures, ATC codes, target sequences, semantic sim.(GO), pathways	drug-disease assoc., disease-gene assoc., semantic sim.(GO), PPIs	protein sequences, semantic sim.(GO), PPIs
Deep Learning	deepDR [38]	MDA, cVAE	DDIs, DTIs, chemical structures, target sequences, semantic sim.(GO), side effects, etc.	-	-
	ANMF [39]	autoencoder	chemical structures	MimMiner	-
	NEDD [40]	HIN2vec	chemical structures	MimMiner	-
	SNF-NN [41]	SNF, neural networks	chemical structures, DTIs, side effects	disease-gene assoc., disease-miRNA assoc., phenotypes	-
	SAEROF [42]	autoencoder, rotation forest	chemical structures	semantic sim.(MeSH)	-
	LAGCN [43]	GCN	target features, chemical structures, DDIs, pathways, etc.	semantic sim.(MeSH)	-

**Table 2:** Number of distinct drugs, diseases, genes, and edges in the tripartite graph created based on the associations between different types of nodes in the experiment.

	Associations	Number of Drugs	Number of Diseases	Number of Genes	Number of Edges
Network-CS	Drug–Disease	615	285	-	1728
	Drug–Gene	450	-	14,430	170,652
	Disease–Gene	-	180	323	390
Network-ATC	Drug–Disease	593	282	-	1681
	Drug–Gene	437	-	14,430	167,535
	Disease–Gene	-	180	323	390

**Table 3:** AUC, AUPR, and AUPR* of drug–disease association prediction results using network-CS. Values close to the highest are expressed in bold.

	Method	Prediction on the Drug Side			Prediction of the Disease Side		
		AUC	AUPR	AUPR*	AUC	AUPR	AUPR*
Graph Mining	MBiRW	0.753	0.046	0.762	0.692	**0.195**	0.769
	TP-NRWRH	0.746	0.043	0.753	**0.751**	0.084	**0.795**
	DR-IBRW	0.747	0.046	0.758	0.704	**0.162**	0.776
	BGMSDDA	0.790	0.078	0.804	0.694	0.089	0.760
Matrix Factorization/ Matrix Completion	DRRS	0.761	0.048	0.768	0.731	0.081	0.763
	OMC	**0.813**	0.076	0.820	**0.751**	0.029	0.715
	DRIMC	0.749	0.051	0.764	0.700	0.051	0.732
	MSBMF	**0.805**	**0.176**	**0.842**	0.669	0.048	0.708
Deep Learning	deepDR	0.685	0.024	0.686	0.606	0.016	0.613
	ANMF	0.646	0.030	0.678	0.673	0.037	0.692
	LAGCN	0.751	0.042	0.756	0.643	0.031	0.677

**Table 4:** AUC, AUPR, and AUPR* of drug–disease association prediction results using network-ATC. Values close to the highest are expressed in bold.

	Method	Prediction on the Drug Side			Prediction of the Disease Side		
		AUC	AUPR	AUPR*	AUC	AUPR	AUPR*
Graph Mining	MBiRW	**0.893**	**0.390**	**0.917**	0.768	**0.207**	0.819
	TP-NRWRH	0.840	0.140	0.855	0.775	0.090	0.809
	DR-IBRW	0.853	0.309	0.887	0.720	0.174	0.786
	BGMSDDA	**0.881**	0.340	0.701	0.705	0.139	0.765
Matrix Factorization/ Matrix Completion	DRRS	**0.890**	0.291	**0.909**	0.755	0.117	0.796
	OMC	0.852	**0.343**	0.883	**0.813**	**0.214**	**0.845**
	DRIMC	0.807	0.080	0.820	0.699	0.043	0.726
	MSBMF	0.872	0.300	0.902	0.702	0.053	0.735
Deep Learning	deepDR	0.730	0.028	0.714	0.614	0.017	0.620
	ANMF	0.845	0.199	0.868	0.739	0.050	0.743
	LAGCN	0.842	0.079	0.843	0.742	0.044	0.753

**Table 5:** AUC of drug–disease association prediction results using a gold standard dataset, and the increasing or decreasing rates of the AUC by removing and adding 10% and 20% of drug–disease associations, respectively.

	Method	Edge Removal		AUC	Edge Addition	
		−20%	−10%		10%	20%
Graph Mining	MBiRW	−1.47%	0.16%	0.884	−0.41%	−2.48%
	TP−NRWRH	−3.39%	−1.90%	0.923	−2.24%	−4.34%
	DR−IBRW	−4.93%	−0.26%	0.871	−1.52%	−5.06%
	BGMSDDA	−4.45%	−3.28%	0.832	0.10%	−1.55%
Matrix Factorization/ Matrix Completion	DRRS	−2.93%	−0.37%	0.913	−1.19%	−2.27%
	OMC	−3.61%	4.86%	0.941	−1.18%	−2.67%
	DRIMC	−2.78%	−0.91%	0.878	−0.44%	−2.80%
	MSBMF	−3.31%	−1.20%	0.901	−0.59%	−2.56%
Deep Learning	deepDR	−4.80%	−3.44%	0.850	−2.53%	−4.21%
	ANMF	−4.81%	−0.75%	0.935	−1.64%	−1.82%
	LAGCN	−1.00%	−1.00%	0.718	−0.21%	−1.00%

## Data Availability

Not applicable.

## Abbreviations

The following abbreviations are used in this manuscript:

CS	Chemical structures
ATC	Anatomic Therapeutic Chemical
PPI	Protein-protein interaction
DTI	Drug-target interaction
ROC	Receiver operating characteristic
AUC	The area under the ROC curve
AUPR	The area under the precision-recall curve
TP	True positives
TPR	True positive Rate
FP	False positives
FPR	False positive Rate
